# Myeloid-related protein 8 induces self-tolerance and cross-tolerance to bacterial infection via TLR4- and TLR2-mediated signal pathways

**DOI:** 10.1038/srep13694

**Published:** 2015-09-02

**Authors:** Andrew P. Coveney, Wei Wang, Justin Kelly, Jing Hua Liu, Siobhan Blankson, Qiong Di Wu, H. Paul Redmond, Jiang Huai Wang

**Affiliations:** 1 Department of Academic Surgery, University College Cork/National University of Ireland, Cork University Hospital, Cork, Ireland; 2 Department of Pathophysiology, Southern Medical University, Guangzhou 510515, China

## Abstract

Myeloid-related protein 8 (Mrp8) is the active component of Mrp8/14 protein complex released by phagocytes at the site of infection and stimulates inflammatory responses. However, it is unclear whether Mrp8 could induce self-tolerance and cross-tolerance to bacterial infection. Here we report that Mrp8 triggered TNF-α and IL-6 release via a Toll-like receptor 4 (TLR4)-dependent manner. Pre-stimulation of murine macrophages and human monocytes with Mrp8 induced self-tolerance to Mrp8 re-stimulation and cross-tolerance to lipopolysaccharide (LPS), bacterial lipoprotein (BLP), gram-negative and gram-positive bacterial challenges, with substantially attenuated TNF-α and IL-6 release. Moreover, Mrp8 tolerisation significantly reduced serum TNF-α and IL-6, increased polymorphonuclear neutrophil (PMN) recruitment and accelerated bacterial clearance, thus protecting mice against LPS-induced lethality and cecal ligation and puncture (CLP)-induced polymicrobial sepsis. In addition to TLR4, TLR2 also contributed to Mrp8-induced inflammatory response and tolerance. Down-regulation of phosphorylated p38 by Mrp8 pre-stimulation was predominantly responsible for the intracellular mechanism of Mrp8-induced tolerance. Thus, our findings of Mrp8-induced self-tolerance and cross-tolerance may provide a potential strategy for attenuating an overwhelming proinflammatory cascade and enhancing antimicrobial responses during microbial sepsis.

Tissue damage caused by trauma, infection or inflammation is associated with the release of endogenous proteins that signal impending danger to the host. The terms “damage-associated molecular patterns (DAMPs)” or “alarmins” have been used to collectively describe endogenous proteins that signal tissue and cell damage which may be present in the absence of microbial pathogens^1^. These molecules help to explain the initiation of an inflammatory response in the absence of infection such as in trauma or the classical example of acute pancreatitis. Endogenous alarmins and exogenous pathogen-associated molecular patters (PAMPs) convey a similar message and elicit similar inflammatory responses, and can therefore be considered subgroups of a large set of molecules called “DAMPs”. There is an ever increasing list of endogenous intracellular components that have been found to initiate inflammation and act as alarmins. These include proteins such as high mobility group box 1 (HMGB1), HSP70, uric acid, the S100 proteins and cellular RNA released during cellular injury. These have all been found to induce Toll-like receptor (TLR)-dependent inflammatory response^1^,^2^,^3^,^4^,^5^,^6^,^7^. Significantly, some of these molecules, including HMGB1 and HSP70 are not released during apoptosis, which is in keeping with the idea that programmed cell death does not result in an inflammatory response^8^,^9^,^10^,^11^.

The S100 proteins represent a group of more than twenty calcium-binding proteins, and three of which, also known as myeloid-related proteins (Mrps) or calgranulins, have been linked to innate immune functions by their expression on myeloid cells. The most prominent of these are S100A8 (Mrp8), S100A9 (Mrp14) and S100A12 (Mrp6). Of these, Mrp8 and Mrp14 form a heterodimer, which is the biological relevant form of these proteins^7^,^12^,^13^,^14^. The Mrp8/14 complex induces a variety of inflammatory reactions and the level of Mrp8/14 expression correlates with disease activity in several autoimmune inflammatory disorders including rheumatoid arthritis, SLE and inflammatory bowel disease, and is also elevated during infection^13^,^15^,^16^. Mrp8/14 is involved in the recruitment of inflammatory cells to the site of injury^16^,^17^. In patients with peritonitis, Mrp8/14 levels in their abdominal fluid were found to be more than 15-fold higher than in their plasma^18^, supporting the theory that the Mrp8/14 complex is released at the site of injury or infection. Mrp8 and Mrp14 are both secreted by phagocytes at sites of inflammation^16^ and both molecules have proinflammatory effects^19^,^20^. They are secreted by a non-classical pathway^19^ and represent the most abundant cytoplasmic proteins in neutrophils and monocytes^15^,^17^,^21^. Extracellularly, Mrp8, Mrp14 and the Mrp8/14 complex all mediate regulatory and biological functions including anti-proliferative, anti-tumoral, anti-microbial and anti-nociceptive activities^22^,^23^,^24^,^25^,^26^,^27^. There is a strong association between the Mrp8/14 complex and its use as a potential novel biomarker in multiple inflammatory disorders including inflammatory bowel disease^28^,^29^, atheroscelerosis^30^, vasculitis^31^, SLE^32^ and rheumatoid arthritis^33^,^34^.

Mrp8/14 is an endogenous activator of TLR4, promoting lethal endotoxin-induced shock. It has been shown that Mrp8/14 represents a molecular system involved in the pathogenesis of septic shock^7^. Mrp8/14 was found to amplify lipopolysaccharide (LPS)-induced TNF-α release *in vitro* and *in vivo*, and mice with targeted deletion of the *Mrp14* gene were protected against LPS-induced lethal shock. Mrp8 is almost not detectable at the protein level in mature phagocytes of Mrp14-deficient mice despite normal Mrp8 mRNA levels, probably due to an elevated metabolism of Mrp8 in the absence of its binding partner Mrp14. Thus, targeted deletion of *Mrp14* leads to a complete lack of a functional Mrp8/14 complex in mice^7^,^35^,^36^. Mrp8 was found to be the active component of the Mrp8/14 complex that induces the translocation of myeloid differentiation factor 88 (MyD88) and activations of IL-1 receptor associated kinase-1 (IRAK-1) and NF-κB, resulting in an elevated expression of TNF-α^7^. An increased inflammatory response may represent more of a danger to the host rather than protection against bacterial infection, as seen in the setting of septic shock. This was confirmed when Mrp14-deficient mice showed an improved survival after the intraperitoneal injection of live *E. coli*^7^.

Taking into account the high levels of Mrp8/14 in inflammatory disease and infection, immune intervention that targets this complex may be a successful strategy to block an uncontrolled inflammatory response seen in severe sepsis and septic shock. The inhibition of release of this inflammatory mediator may represent a promising therapeutic option. Alternatively, using a low dose of Mrp8 to induce a state of immune “tolerance” to subsequent inflammatory stimuli, as seen in the case of the TLR4 agonist LPS, may represent another viable therapeutic option. In the present study we attempted to investigate the potential role of Mrp8 in inducing self-tolerance and cross-tolerance to bacterial infection.

## Results

### Mrp8 dose- and time-dependently induces self-tolerance and cross-tolerance in murine macrophages

**Figure 1:**
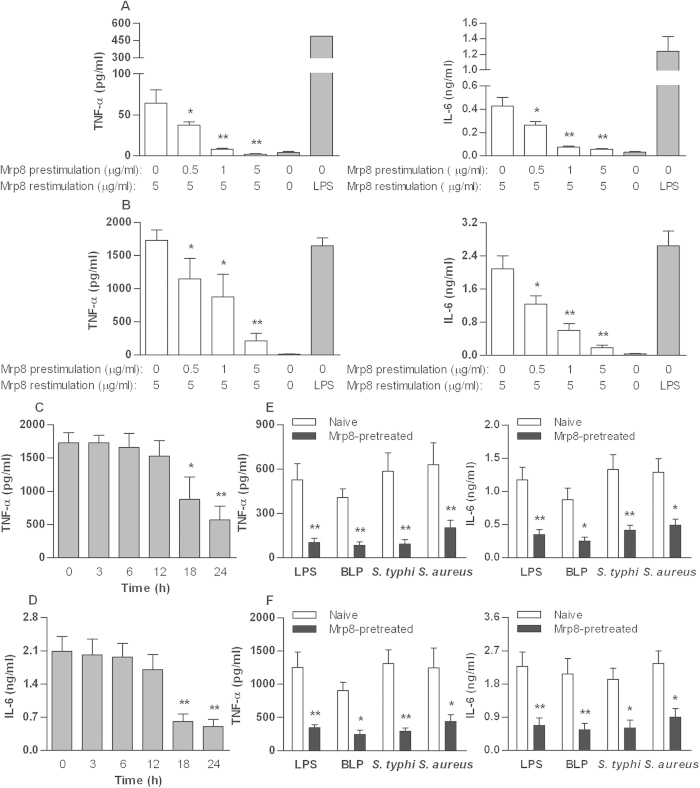
Mrp8 dose- and time-dependently induces self-tolerance and cross-tolerance in murine macrophages. Peritoneal macrophages (**A**) and BMMs (**B**) isolated from C3H/HeN mice were pre-stimulated with increasing doses of mMrp8 for 18 h and re-stimulated with 5 μg/ml mMrp8 for 6 h. Naïve murine macrophages stimulated with 100 ng/ml LPS (**A**,**B**) for 6 h were used as the positive control. (**C**,**D**) Murine BMMs were pre-stimulated with 1 μg/ml mMrp8 for the indicated time periods and re-stimulated with 5 μg/ml mMrp8 for 6 h. Murine peritoneal macrophages (**E**) and BMMs (**F**) were pre-incubated with either culture medium (naive) or 1 μg/ml mMrp8 (Mrp8-pretreated) for 18 h and re-stimulated with 100 ng/ml LPS, 100 ng/ml BLP, 5 × 10^4^ CFU/ml heat-killed *S. typhimurium* (*S. typhi*) or 7.5 × 10^4^ CFU/ml heat-killed *S. aureus* for 6 h. TNF-α and IL-6 concentrations in the culture supernatants were assessed by ELISA. Data are presented as mean ± SD of three independent experiments and each experiment was carried out in triplicate. *p < 0.05, **p < 0.01 compared with either naive cells stimulated with 5 μg/ml mMrp8 (**A**–**D**) or naive cells (**E**,**F**).

Stimulation of naïve murine peritoneal macrophages (Fig. 1A) or bone marrow-derived macrophages (BMMs) (Fig. 1B) with murine Mrp8 (mMrp8) for 6 h caused an inflammatory response quantified by significant release of TNF-α and IL-6 compared with macrophages incubated with culture medium alone. Pre-stimulation of murine peritoneal macrophages or BMMs with mMrp8 attenuated the release of TNF-α and IL-6 to a subsequent mMrp8 re-stimulation (Fig. 1A,B). A minimum of 18 h pre-stimulation was required to achieve statistically significant attenuation in TNF-α (Fig. 1C) and IL-6 (Fig. 1D) release. The pre-stimulating effect of mMrp8 was also dose-dependent, with increasing doses of mMrp8 pre-stimulant leading to a greater attenuation in either TNF-α or IL-6 release (Fig. 1A,B). Cell viability was not altered by mMrp8 pre-stimulation as confirmed by Resazurin assay (Supplementary Fig. 1A,B), excluding this as a possible cause for the reduced TNF-α and IL-6 release in mMrp8 pre-stimulated murine macrophages.

Next, we examined whether Mrp8 has an ability to induce cross-tolerance to bacterial infection. Murine peritoneal macrophages (Fig. 1E) or BMMs (Fig. 1F) were pre-incubated with mMrp8 for 18 h and re-stimulated with LPS, bacterial lipoprotein (BLP), or heat-killed bacteria for 6 h. Pre-stimulation with mMrp8 attenuated the release of TNF-α and IL-6 from murine macrophages re-stimulated with LPS or BLP (*p* < 0.05 and *p* < 0.01 versus naive cells) (Fig. 1E,F), confirming mMrp8-induced cross-tolerance to both TLR4 (LPS) and TLR2 (BLP) agonists in murine macrophages. Furthermore, mMrp8 also demonstrated the induction of cross-tolerance to bacterial challenges, as significantly attenuated release of TNF-α and IL-6 in response to heat-killed gram-negative *Salmonella typhimurium* (*S. typhimurium*) or heat-killed gram-positive *Staphylococcus aureus* (*S. aureus*) was observed in mMrp8-prestimulated murine macrophages (*p* < 0.05 and *p* < 0.01 versus naïve cells) (Fig. 1E,F).

### Human monocytes demonstrate Mrp8 self-tolerance and cross-tolerance to LPS, BLP and bacteria

**Figure 2:**
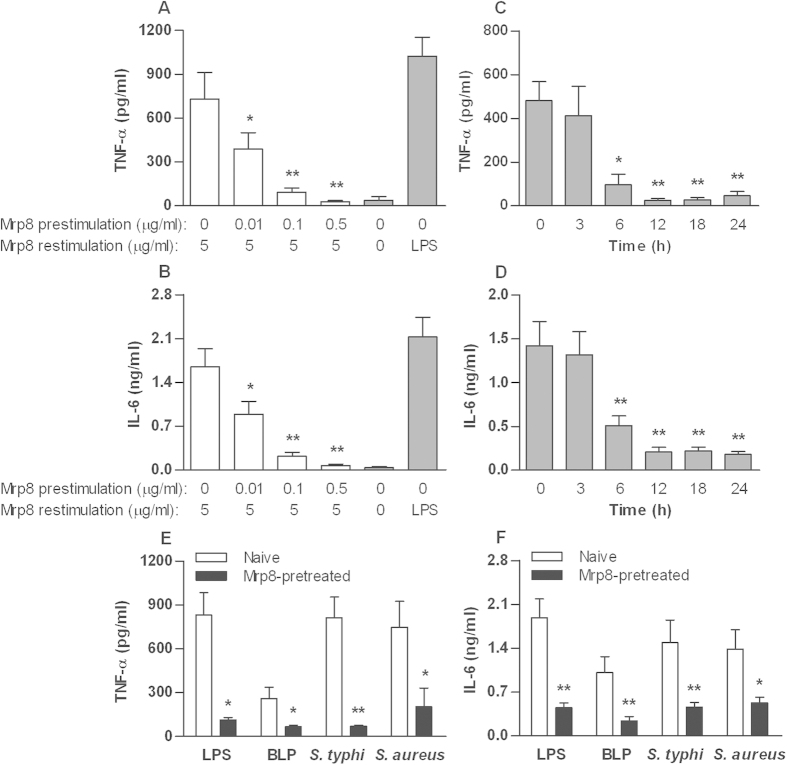
Mrp8 induces self-tolerance and cross-tolerance to LPS, BLP and bacteria in human monocytes. Isolated human monocytes were pre-stimulated with increasing doses of hMrp8 for 12 h and re-stimulated with 5 μg/ml hMrp8 for 6 h (**A**,**B**) or pre-stimulated with 0.1 μg/ml hMrp8 for the indicated time periods and re-stimulated with 5 μg/ml hMrp8 for 6 h (**C**,**D**). Naïve human monocytes were also stimulated with 100 ng/ml LPS (**A**,**B**) for 6 h as the positive control. (**E**,**F**) Human monocytes were pre-incubated with either culture medium (naive) or 0.1 μg/ml hMrp8 (Mrp8-pretreated) for 12 h and re-stimulated with 100 ng/ml LPS, 100 ng/ml BLP, 5 × 10^4^ CFU/ml heat-killed *S. typhimurium* (*S. typhi*) or 7.5 × 10^4^ CFU/ml heat-killed *S. aureus* for 6 h. TNF-α and IL-6 concentrations in the culture supernatants were assessed by ELISA. Data are presented as mean ± SD of three independent experiments and each experiment was carried out in quadruplicate. *p < 0.05, **p < 0.01 compared with either naive cells stimulated with 5 μg/ml hMrp8 (**A**–**D**) or naive cells (**E**,**F**).

As with murine macrophages, human Mrp8 (hMrp8) stimulation of human monocytes isolated from healthy volunteers led to significant release of TNF-α (Fig. 2A) and IL-6 (Fig. 2B), confirming the inflammatory effect of hMrp8 on human monocytes. Human monocytes were pre-stimulated with different doses of hMrp8 and re-stimulated with 5 μg/ml hMrp8 to investigate for the self-tolerising effect previously seen in murine macrophages. Human monocytes appeared to be significantly more sensitive to the tolerising effect of hMrp8 compared with murine macrophages. Induction of Mrp8 tolerance in human monocytes was found to be both dose- and time-dependent as seen in murine macrophages, but required smaller Mrp8 doses (Fig. 2A,B) and shorter pre-stimulation periods (Fig. 2C,D) in comparison to murine macrophages. Human monocytes only required 6 h of pre-stimulation with hMrp8 to induce significant self-tolerance which represents a third of the pre-stimulation period required in murine macrophages. The pre-stimulation dose as low as 0.01 μg/ml of hMrp8 was found to significantly attenuate the TNF-α and IL-6 release from human monocytes. In addition, the viability of human monocytes was not altered by hMrp8 pre-stimulation as measured by Resazurin assay (Supplementary Fig. 1C).

Pre-stimulation of human monocytes with hMrp8 also resulted in an attenuated inflammatory response to either LPS or BLP re-stimulation (*p* < 0.05 and *p* < 0.01 versus naive cells) (Fig. 2E,F). Moreover, a significant attenuation in either TNF-α (Fig. 2E) or IL-6 (Fig. 2F) release was observed in hMrp8 pre-stimulated human monocytes after re-stimulation with heat-killed *S. typhimurium* or *S. aureus* (*p* < 0.05 and *p* < 0.01 versus naive cells), demonstrating that similar to mMrp8, hMrp8 pre-stimulation induces cross-tolerance to bacterial infection in human monocytes also.

We further used polymyxin B, a specific LPS inhibitor, to ascertain that Mrp8-induced inflammatory response and tolerance was not due to the contamination of LPS. Polymyxin B at 25 μg/ml efficiently blocked LPS-stimulated inflammatory response but had no effect on TNF-α release from mMrp8-stimulated murine BMMs (Supplementary Fig. 2A). By contrast, heat-treated mMrp8, but not heat-treated LPS, lost its stimulatory effect on murine BMMs (Supplementary Fig. 2A). Similar results were also found in human monocytes stimulated either with hMrp8 in the presence of polymyxin B or with heat-inactivated hMrp8 (Supplementary Fig. 2B). These results clearly rule out any effects from a potential LPS contamination of the Mrp8 preparation.

### Mrp8-mediated inflammatory response and tolerance are both TLR4- and TLR2-dependent

**Figure 3:**
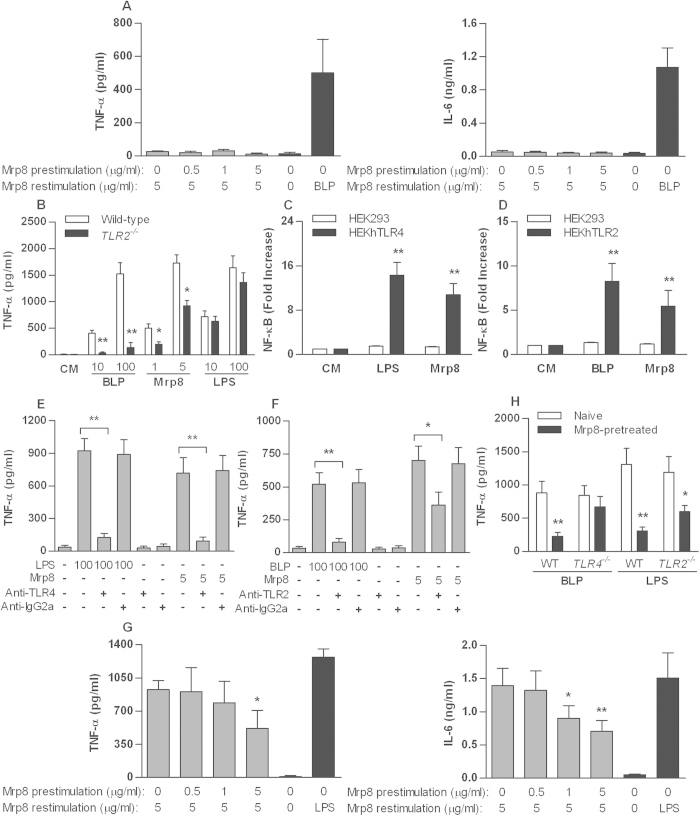
Both TLR4 and TLR2 are involved in Mrp8-mediated inflammatory response and tolerance. BMMs isolated from TLR4-deficient (**A**) or TLR2-deficient (**G**) mice were pre-stimulated with increasing doses of mMrp8 for 18 h and re-stimulated with 5 μg/ml mMrp8 for 6 h. Naïve murine BMMs were also stimulated with 100 ng/ml BLP (**A**) or 100 ng/ml LPS (**G**) for 6 h as the positive control. (**B**) BMMs isolated from wild-type and TLR2-deficient mice were stimulated with culture medium (CM), 1 and 5 μg/ml mMrp8, 10 and 100 ng/ml BLP or 10 and 100 ng/ml LPS for 6 h. HEK293 and HEKhTLR4 cells (**C**) or HEK293 and HEKhTLR2 cells (**D**) transfected with the NF-κB promoter luciferase plasmid were stimulated with CM, 5 μg/ml hMrp8, 100 ng/ml LPS or 100 ng/ml BLP for 6 h. (**E**,**F**) Human monocytes were pre-incubated with 2 μg/ml of either the anti-TLR4 mAb (HTA125), anti-TLR2 mAb (TL2.1) or an isotype control mAb (IgG2a) for 30 min and further stimulated with 5 μg/ml hMrp8, 100 ng/ml LPS or 100 ng/ml BLP for 6 h. (**H**) BMMs isolated from wild-type, TLR4-deficient or TLR2-deficient mice were pre-incubated with either CM (naive) or 1 μg/ml mMrp8 (Mrp8-pretreated) for 18 h and re-stimulated with 100 ng/ml BLP or 100 ng/ml LPS for 6 h. TNF-α and IL-6 concentrations in the culture supernatants were assessed by ELISA. NF-κB activation was expressed as fold increases of luciferase activity relative to HEK293, HEKhTLR4 or HEKhTLR2 cells incubated with CM. Data are expressed as mean ± SD of three to four independent experiments and each experiment was conducted in triplicate. *p < 0.05, **p < 0.01 compared with wild-type BMMs (**B**), HEK293 cells (**C**,**D**), human monocytes pre-incubated with anti-TLR4 (**E**) or anti-TLR2 (**F**) blocking mAbs, naive cells stimulated with 5 μg/ml mMrp8 (**G**) or naive cells (**H**).

To ascertain whether Mrp8-induced inflammatory response and tolerance are dependent on either TLR4 or TLR2, naïve and mMrp8 pre-stimulated TLR4- and TLR2-deficient murine macrophages were re-stimulated with mMrp8. In the absence of TLR4, mMrp8 failed to elicit an inflammatory response (Fig. 3A), confirming that Mrp8-induced inflammatory response acts via TLR4. However, naïve TLR2-deficient macrophages displayed a blunted TNF-α release to BLP stimulation but a normal TNF-α response to LPS stimulation, also showed an attenuated TNF-α release to mMrp8 stimulation relative to wild-type macrophages (*p* < 0.05) (Fig. 3B). Stimulation of HEKhTLR4 cells with hMrp8 led to a strong NF-κB activation compared with HEK293 cells (*p* < 0.01) (Fig. 3C), whereas an enhanced NF-κB activation was also observed in hMrp8-stimulated HEKhTLR2 cells (*p* < 0.01 versus hMrp8-stimulated HEK293 cells) (Fig. 3D). A specific anti-TLR4 mAb almost completely blocked the stimulatory effect of hMrp8 on TNF-α response (Fig. 3E), and interestingly, a specific anti-TLR2 mAb also significantly attenuated hMrp8-stimulated TNF-α release from human monocytes (*p* < 0.05) (Fig. 3F). These results suggest that, in addition to TLR4, TLR2 also contributes to Mrp8-induced inflammatory response.

Pre-stimulation of TLR2-deficient macrophages with mMrp8 demonstrated less attenuation in TNF-α and IL-6 release to mMrp8 re-stimulation (Fig. 3G) relative to that previously seen in wild-type macrophages (Fig. 1B), suggesting the involvement of TLR2 in Mrp8-induced self-tolerance. To further clarify the impact of TLR4 and TLR2 on Mrp8-mediated inflammatory response and tolerance, we either pretreated TLR4-deficient macrophages with mMrp8 and re-stimulated these cells with the TLR2 agonist BLP or pretreated TLR2-deficient macrophages with mMrp8 and re-stimulated these cells with the TLR4 agonist LPS. In the absence of TLR4, mMrp8-induced cross-tolerance to BLP was almost completely abrogated in TLR4-deficient macrophages, whereas in the absence of TLR2, mMrp8-induced cross-tolerance to LPS was still observed in TLR2-deficient macrophages but was substantially diminished compared with mMrp8-pretreated wild-type macrophages (Fig. 3H). Collectively, these results indicate that TLR4 may function as the predominant receptor for Mrp8; however, TLR2 appears to be also involved in Mrp8-induced inflammatory response and tolerance.

### The attenuated inflammatory effect of Mrp8 pre-stimulation is primarily through the p38 pathway rather than the NF-κB pathway

To further investigate the intracellular mechanism involved in the attenuated inflammatory response during Mrp8 self-tolerance and cross-tolerance, we assessed two downstream intracellular signal transduction components p38 and the inhibitor of κBα (IκBα) common to both the TLR2 and TLR4 signalling pathways. The phosphorylated version of p38 has been shown to accurately measure p38 activity^37^. IκBα inhibits the transcription factor NF-κB by retaining it in the cytoplasm. Upon activation of the NF-κB pathway, IκBα is rapidly phosphorylated and degradated, which liberates NF-κB. Activation of NF-κB therefore correlates with phosphorylation of IκBα and requires proteolysis of this inhibitor^38^.

**Figure 4:**
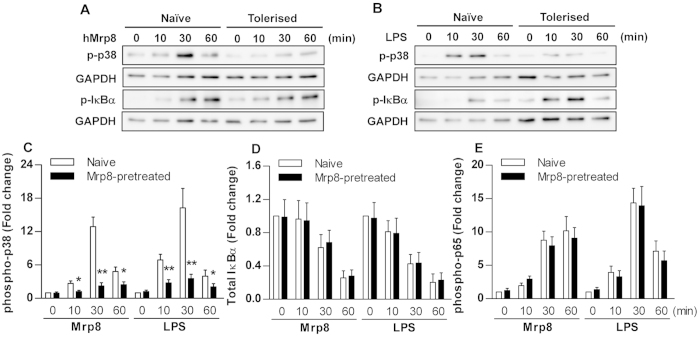
Mrp8 pre-stimulation attenuates p38 phosphorylation in human monocytes re-stimulated with either Mrp8 or LPS. Isolated human monocytes were pre-stimulated with either culture medium (naive) or 0.1 μg/ml hMrp8 (Mrp8-tolerised) for 12 h and re-stimulated with 5 μg/ml hMrp8 (**A**) and 10 ng/ml LPS (**B**) or 5 μg/ml hMrp8 and 100 ng/ml LPS (**C**–**E**) for the indicated time periods. (**A**,**B**) Cytoplasmic proteins were extracted and subjected to immunoblotting for detection of phosphorylated p38 (p-p38) and phosphotylated IκBα (p-IκBα). Results shown represent one experiment from a total of three separate experiments. (**C**–**E**) human monocytes were fixed, permeabilized and stained with Alexa Fluor 488- or PE-conjugated anti-IκBα, anti-phospho-p65 or anti-phopho-p38 mAbs. Total IκBα protein and phosphorylation of p65 and p38 were detected by FACScan analysis and expressed as fold changes of MFI. Data are presented as mean ± SD of four independent experiments and each experiment was conducted in duplicate. *p < 0.05, **p < 0.01 compared with naive cells.

As shown in Fig. 4A, expression of the phosphorylated p38 increased rapidly within an hour of hMrp8 stimulation in naïve human monocytes. Pre-stimulation of human monocytes with hMrp8 led to a substantial reduction in phosphorylated p38 at 30 and 60 min after re-stimulation with hMrp8 compared with naïve cells. Levels of the phosphorylated IκBα also increased rapidly after stimulation with hMrp8 in naive cells; however, pre-stimulation of human monocytes with hMrp8 did not attenuate IκB-α phosphorylation in response to hMrp8 re-stimulation (Fig. 4A). The same experiment was repeated using LPS as the re-stimulant to investigate the intracellular mechanism during hMrp8-induced cross-tolerance. Again, phosphorylation of p38 in response to LPS re-stimulation was downregulated in hMrp8-tolerised human monocytes compared with naïve cells, but IκBα phosphorylation was unaltered or even slightly enhanced by hMrp8 pre-stimulation (Fig. 4B).

We further assessed levels of total IκBα, phosphorylated NF-κB p65 and p38 by FACScan analysis in naive and hMrp8-prestimulated human monocytes after re-stimulation with hMrp8 or LPS. Stimulation of naive human monocytes with either hMrp8 or LPS resulted in a strong activation of p38, whereas hMrp8 pre-stimulation significantly attenuated both hMrp8- and LPS-induced p38 phosphorylation (*p* < 0.05 and *p* < 0.01 versus naive cells) (Fig. 4C). Stimulation with hMRP8 or LPS caused IκBα degradation as represented by substantially reduced total IκBα protein and activated NF-κB p65 in naive cells; however, pre-stimulation with hMrp8 failed to prevent hMrp8- or LPS-induced IκBα degradation (Fig. 4D) and NF-κB p65 phosphorylation (Fig. 4E). These results indicate that the attenuated inflammatory effect of Mrp8 pre-stimulation is primarily through the p38 MAPK pathway rather than the NF-κB pathway during Mrp8-induced both self-tolerance and cross-tolerance.

### Mrp8 tolerisation affords protection against both LPS-induced lethality and CLP-induced polymicrobial sepsis

**Figure 5:**
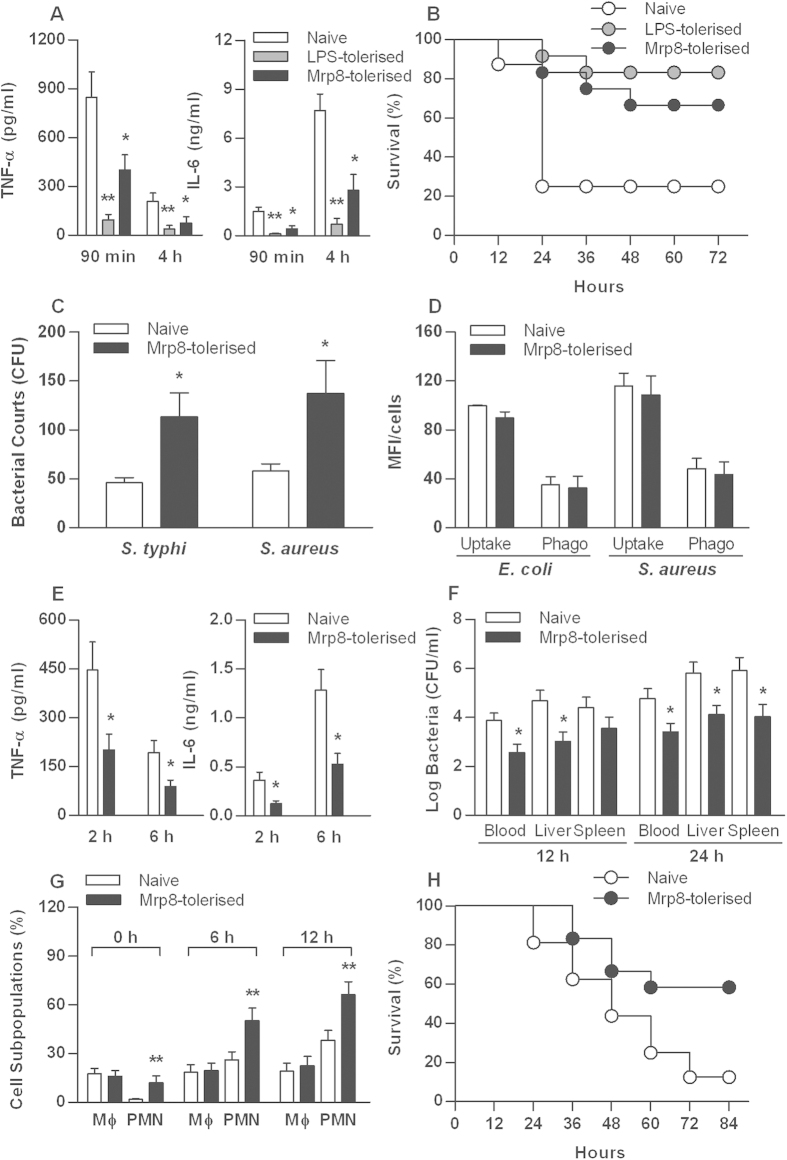
Mrp8 pretreatment protects mice against LPS-induced lethality and CLP-induced polymicrobial sepsis. C3H/HeN mice pretreated with PBS (naive), 5 mg/kg LPS (LPS–tolerised) or 50 μg/mouse mMrp8 (Mrp8-tolerised) for 24 h were challenged with 30 mg/kg LPS (**A**,**B**) or subjected to CLP-induced polymicrobial infection (**E**–**H**). Serum TNF-α and IL-6 either at 90 min and 4 h post LPS challenge (**A**) or at 2 and 6 h post CLP (**E**) were assessed by cytometric bead array. Data are shown as mean ± SD from four mice per time point and representative of three separate experiments. *p < 0.05, **p < 0.01 compared with naive mice. Kaplan-Meier survival curve shows significantly improved survival in LPS-tolerised mice (n = 12) (*p* = 0.0021) and mMrp8-tolerised mice (n = 12) (*p* = 0.0153) compared to naive mice (n = 16) after lethal LPS challenge (**B**) or in mMrp8-tolerised mice (n = 12) (*p* = 0.0202) compared to naive mice (n = 16) after CLP-induced polymicrobial infection (**H**). Bacterial clearance from the blood and visceral organs collected at 12 and 24 h post CLP (**F**) was expressed as log CFU/ml, and subpopulations of PMNs and macrophages in the peritoneal exudate before and after CLP (**G**) were analyzed by flow cytometry. Data are shown as mean ± SD from six mice per time point and representative of three separate experiments. *p < 0.05, **p < 0.01 compared with naive mice. Human monocytes were pre-stimulated with culture medium (naive) or 0.1 μg/ml hMrp8 (Mrp8-tolerised) for 12 h, and further incubated either with live *S. typhimurium* (*S. typhi*), live *S. aureus* for 50 min to assess intracellular bacterial killing (**C**) or with FITC-conjugated *E. coli*, FITC-conjugated *S. aureus* for 30 min to assess bacterial uptake and phagocytosis (**D**). Bacterial counts, bacterial uptake and phagocytosis were expressed as CFU and MFI per cell. Data are presented as mean ± SD of three independent experiments and each experiment was conducted in quadruplicate. *p < 0.05 compared with naive cells.

We pretreated mice for 24 h either with a low dose of LPS (5 mg/kg) to induce LPS tolerance or with mMrp8 (50 μg/mouse) and further challenged these mice with a lethal dose of LPS (30 mg/kg). We analyzed serum levels of TNF-α and IL-6 in naive, LPS-pretreated and mMrp8-pretreated mice. LPS tolerisation almost completely attenuated TNF-α and IL-6 release in LPS-challenged mice (Fig. 5A). Pre-treatment with mMrp8 also led to a significant reduction in peak serum levels of TNF-α at 90 min and IL-6 at 4 h post LPS challenge (*p* < 0.05 versus naive mice) (Fig. 5A), demonstrating that mMrp8 is capable of inducing cross-tolerance to LPS *in vivo*. Moreover, mMrp8 pre-treatment protected mice from LPS-induced lethality, with a significant decrease in mortality from 75% in naive mice to 33% in mMrp8-pretreated mice after lethal LPS challenge (*p* = 0.0153) (Fig. 5B). This was similar to the survival advantage observed in LPS-tolerised mice (*p* = 0.0021 versus naive mice) (Fig. 5B).

We next compared intracellular bacterial killing by naïve and hMrp8-tolerised human monocytes, and found that hMrp8-tolerised human monocytes displayed a higher intracellular load of either *S. typhimurium* or *S. aureus* compared with naive cells (*p* < 0.05) (Fig. 5C). While an increase in intracellular bacterial load is most likely explained by a reduction in intracellular killing of the ingested bacteria, it could also be associated with a change in the rate of bacterial uptake and phagocytosis. To examine this possibility, we assessed uptake and phagocytosis of FITC-labeled *E. coli* and *S. aureus* by naive and hMrp8-tolerised human monocytes. There were no significant differences seen in uptake and phagocytosis of either *E. coli* or *S. aureus* between naïve and hMrp8-tolerised monocytes (Fig. 5D), suggesting that the increased intracellular bacterial load is due to reduced intracellular killing of the ingested bacteria in hMrp8-tolerised human monocytes.

As hMrp8-tolerised human monocytes exhibited reduced intracellular bacterial killing, we further examined whether Mrp8 tolerisation could render mice more susceptible to bacterial infection. Naive and mMrp8-tolerised mice were subjected to cecal ligation and puncture (CLP)-induced polymicrobial infection. Similar to a substantially attenuated proinflammatory response to LPS challenge seen in mMrp8-tolerised mice, mMrp8 tolerisation significantly diminished serum peak levels of TNF-α at 2 h and IL-6 at 6 h post CLP (*p* < 0.05 versus naive mice) (Fig. 5E). Significantly reduced bacterial counts were observed in the blood and visceral organs of mMrp8-tolerised mice at 12 h and 24 h post CLP (*p* < 0.05 versus naive mice) (Fig. 5F), indicating that mMrp8-tolerised mice display accelerated bacterial clearance in response to polymicrobial infection. As polymorphoneuclear neutrophil (PMN) influx from the circulation into the infectious site during bacterial infection plays a key role in eradicating the invaded microbial pathogens^39^, we measured leukocyte subpopulations in the peritoneal cavity of naive and mMrp8-tolerised mice before and after being challenged with CLP. As shown in Fig. 5G, mMrp8 tolerisation resulted in an increased PMN accumulation in the peritoneal cavity, and in response to polymicrobial infection, mMrp8 tolerisation recruited significantly more PMNs into the peritoneal cavity (*p* < 0.01 versus naive mice). Consistent with the accelerated bacterial clearance and enhanced PMN recruitment, mMrp8-tolerised mice were more resistant to CLP-induced polymicrobial sepsis with an overall survival of 58% compared with a 12% survival rate in naive mice (*p* = 0.0202) (Fig. 5H).

## Discussion

The innate immune system responds rapidly through activation of pattern recognition receptors (PRRs) such as TLRs that interact with highly conserved molecules present in microorganisms^40^,^41^, and thus plays an increasingly important role in the pathogenesis of sepsis. Binding of TLRs to epitopes on microorganisms stimulates intracellular signalling and increases transcription of proinflammatory molecules such as TNF-α, IL-1β and IL-6 as well as anti-inflammatory cytokines such as IL-10^42^. Both TLR2 and TLR4 are the key PRRs involved in microbial sepsis. TLR4 is the PRR for LPS/ endotoxin, an abundant constituent of the bacterial cell wall of gram-negative bacteria and plays a key role in the pathogenesis of gram-negative sepsis^43^,^44^. TLR2, acting as a heterodimer with TLR1 or TLR6, is the PRR for a number of gram-positive molecules and plays a key role in the pathogenesis of gram-positive sepsis^45^,^46^,^47^. Endogenous proteins known as “DAMPs” which are released during infection or tissue damage have also been shown to activate these same PRRs^1^,^2^,^3^,^4^,^5^,^6^,^7^. Mrp8, which forms the endogenous protein complex Mrp8/14 with Mrp14, has been shown to activate TLR4 and promotes lethal endotoxin-induced shock in mice^7^. In the present study, we demonstrated the proinflammatory effect of mMrp8 on murine macrophages as represented by TNF-α and IL-6 production, and stimulation of human monocytes with hMrp8 resulted in a similar inflammatory response as seen in murine macrophages.

Although Mrp8 was reported to promote lethal endotoxin-induced shock^7^, we proposed that Mrp8 may also offer transient protection for the host against an overactive inflammatory cascade through a tolerance-like process. LPS or endotoxin tolerance provides a protective mechanism for the host against over-exuberant inflammation in the setting of recurrent exposure to endotoxin and improves survival to polymicrobial sepsis in a murine *in vivo* model^48^. LPS tolerance is a phenomenon in which cells or organisms exposed to a low concentration of endotoxin enter into a transient unresponsive state during which they are unable to respond to further challenges with endotoxin^49^. A similar phenomenon is also seen with the TLR2 agonist BLP^50^. Moreover, a recent study has reported that Mrp8 and Mrp14 induce tolerance in neonatal murine phagocytes under sterile inflammatory conditions^51^.

In the present study we found that pre-stimulation of murine macrophages with low doses of mMrp8 attenuated the inflammatory response to a subsequent higher dose of mMrp8. The same result was also seen in human monocytes pre-stimulated with low doses of hMrp8, confirming the self-tolerising effect of Mrp8 in both murine and human *in vitro* models. Furthermore, Mrp8-induced self-tolerising effect was shown to be both dose- and time-dependent in either murine macrophages or human monocytes. Notably, human monocytes were more sensitive to the tolerising effect of Mrp8 with the much smaller Mrp8 pre-stimulating dose and shorter Mrp8 pre-stimulating period than murine macrophages. Reasons for this reduced sensitivity to Mrp8 in murine macrophages compared with human monocytes are unclear, but may be due to evolutionary differences between the two species, where mice are environmentally exposed to greater relative doses of bacteria than humans, thus requiring a much higher pre-stimulating dose to develop tolerance to a subsequent proinflammatory stimulant. The time course we have demonstrated for Mrp8 tolerance is similar to that seen in LPS-tolerance^52^. The time delay required to induce the Mrp8 tolerising effect makes it likely that gene-specific regulatory mechanisms as seen in LPS tolerance^52^ might be also involved in Mrp8-induced tolerance.

Pre-stimulation with Mrp8 was also found to attenuate the inflammatory response to other inflammatory stimuli. In murine macrophages or human monocytes, mMrp8 or hMrp8 pre-stimulation led to significantly attenuated TNF-α and IL-6 release in response to LPS or BLP stimulation, confirming Mrp8-induced cross-tolerance to both TLR4 and TLR2 agonists. These results led us to examine the effect of Mrp8 pre-stimulation on the inflammatory response to bacterial challenges. Pre-stimulation of murine macrophages or human monocytes with mMrp8 or hMrp8 led to a substantially attenuated TNF-α and IL-6 release in response to gram-negative *S. typhimurium* or gram-positive *S. aureus*. Nevertheless, the significant attenuated inflammatory response to LPS, BLP and bacterial stimulation seen in Mrp8-tolerised murine macrophages and human monocytes offers great potential for developing therapeutic strategies during microbial sepsis. Indeed, our *in vivo* results further demonstrated that mice pretreated with mMrp8 for 24 h displayed substantially reduced serum levels of proinflammatory cytokines and significantly improved survival in response to lethal LPS challenge.

TLR4 appears to be the principle PRR for Mrp8, as demonstrated by a nearly completely absent TNF-α and IL-6 response in mMrp8-stimulated TLR4-deficient macrophages and the stimulatory effect of hMrp8 on TNF-α response was almost completely abrogated by a specific anti-TLR4 mAb in human monocytes. However, there was also a relative reduction in TNF-α release from mMrp8-stimulated TLR2-deficient macrophages compared with wild-type macrophages, stimulation with hMrp8 led to a strong NF-κB activation in HEKhTLR2 cells as seen in HEKhTLR4 cells, and a specific anti-TLR2 mAb significantly attenuated hMrp8-stimulated TNF-α release from human monocytes, indicating that TLR2 contributes to Mrp8-stimulated inflammatory response. Notably, the self-tolerising effect of mMrp8 in TLR2-deficient macrophages was substantially diminished with less attenuation in TNF-α and IL-6 release compared with wild-type macrophages. Moreover, while mMrp8-induced cross-tolerance to BLP was almost completely lost in TLR4-deficient macrophages, mMrp8-induced cross-tolerance to LPS was also impaired in TLR2-deficient macrophages. These results suggest that TLR2 is required for Mrp8-induced self-tolerance and cross-tolerance. Taken together, our findings support the notion that in addition to TLR4, TLR2 is also involved in Mrp8-induced inflammatory response and tolerance.

The attenuation of the inflammatory response by Mrp8 pre-stimulation appeared to be primarily at the level of p38, which is one of the downstream intracellular signalling components common to both the TLR4 and TLR2 pathways. Activation of the p38 MAPK pathway plays essential roles in the production of proinflammatory cytokines TNF-α, IL-1β and IL-6^53^. Western blot analysis form the present study demonstrated a substantial reduction of the phosphorylated p38 in hMrp8-tolerised human monocytes re-stimulated with either hMrp8 or LPS. By contrast, hMrp8 pre-stimulation did not attenuate IκBα phosphorylation in either hMrp8 or LPS re-stimulated human monocytes. Consistent with these findings, FACScan analysis further revealed that hMrp8 pre-stimulation significantly downregulated both hMrp8- and LPS-induced p38 phosphorylation, but failed to attenuate hMrp8- or LPS-induced IκBα degradation and NF-κB p65 activation. These results indicate that the p38 MAPK pathway is predominately involved in Mrp8-induced both self-tolerance and cross-tolerance.

In the present study, we found that human monocytes pre-stimulated with hMrp8 showed an increased intracellular bacterial burden in response to bacterial challenge, although there were no differences seen in bacterial uptake and phagocytosis between naïve and hMrp8-tolerised cells. These *in vitro* results indicate a critical possibility of while Mrp8 tolerisation may potentially protect the host from an overwhelming proinflammatory response; it may also predispose the host to bacterial infection. To address this, we challenged naive and mMrp8-tolerised mice with CLP-induced polymicrobial infection. Surprisingly, in addition to significantly attenuated serum TNF-α and IL-6 levels, mMrp8-tolerised mice showed significantly reduced bacterial counts in the circulation and visceral organs in response to polymicrobial infection. PMN influx from the circulation into the infectious site is the critical step to eradicate the invaded microbial pathogens^39^ and successful clearance of bacterial infection has been shown to rely on a rapid and efficient PMN migration into the infectious site such as peritoneal cavity in several experimental established murine polymicrobial sepsis models^54^,^55^,^56^. Therefore, we assessed PMN influx from the circulation into the infectious site in response to polymicrobial infection between naive and mMrp8-tolerised mice and demonstrated that mMrp8 tolerisation recruited substantially more PMNs into the peritoneal cavity, which could well explain the markedly accelerated bacterial clearance observed in mMrp8-tolerised mice. Consequently, mMrp8 tolerisation afforded the protection against polymicrobial sepsis with significantly improved survival in mMrp8-tolerised mice.

In conclusion, we demonstrate that Mrp8 pre-stimulation induces self-tolerance and cross-tolerance to subsequent stimulation with Mrp8, LPS, BLP and gram-negative or gram-positive bacteria in both murine macrophages and human monocytes via a downregulation of the p38 MAPK pathway. Moreover, Mrp8 tolerisation protects mice against LPS-induced lethal shock and CLP-induced polymicrobial sepsis with attenuated proinflammatory cytokine response, enhanced PMN recruitment and accelerated bacterial clearance. These results suggest that Mrp8-induced tolerance may represent a potential therapeutic option during microbial sepsis.

## Methods

### Reagents, Abs, and bacteria

The TLR2 agonist BLP, a synthetic bacterial lipopeptide (Pam_3_-Cys-Ser-Lys_4_-OH) purchased from EMC Microcollections (Tubingen, Germany), was endotoxin-free as confirmed by the *Limulus* amebocyte lysate assay (Charles River Endosafe, Charleston, SC, USA). The TLR4 agonist LPS from *E. coli* serotype O55:B5 was purchased from Sigma-Aldrich (St. Louis, MO, USA). Recombinant mMrp8 and hMrp8 were a gift from Dr. T. Vogl (Institute of Immunology, University of Munster, Munster, Germany) and also purchased from Creative Biomart (Shirley, NY, USA). Mouse anti-human phosphorylated p38 mAb at Tyr^182^ and phosphor- IκBα mAb at Ser^32^ were purchased from Santa Cruz Biotechnology (Santa Cruz, CA, USA) and Cell Signalling (Beverly, MA, USA), respectively. Mouse anti-human TLR4 (HTA125) and TLR2 (TL2.1) blocking mAbs were obtained from Abcam (Cambridge, MA, USA). All culture media and reagents used for cell cultures were purchased from Invitrogen Life Technologies (Paisley, UK). All other chemicals unless stated were purchased from Sigma-Aldrich.

Gram-negative *S. typhimurium* and gram-positive *S. aureus* were obtained from American Type Culture Collection (ATCC, Manassas, VA, USA) and the National University of Ireland Culture Collection, respectively. Bacteria were cultured at 37 °C in trypticase soy broth (Merck, Darmstadt, Germany), harvested at the mid-logarithmic growth phase, washed twice and resuspended in PBS. The concentration of resuspended bacteria was determined and adjusted spectrophotometrically at 550 nm. Both *S. typhimurium* and *S. aureus* were also heat-killed at 95 °C for 20 min.

### Mice, murine macrophage preparation and cultures

Pyrogen-free, 8- to 10-wk-old C3H/HeN (wild-type) and TLR4-deficient (C3H/HeJ) mice were purchased from Harlan (Oxon, UK) and TLR2-deficient mice on the C3H background were a gift from Dr. Carsten J. Kirschning (Institute of Medical Microbiology, Immunology and Hygiene, Technische Universitat Munchen, Munich, Germany). Mice were housed in barrier cages under controlled environmental conditions (12/12 h light/dark cycle, 55 ± 5% humidity, 23 °C) in the University Biological Services Unit, University College Cork. All animal procedures were performed in the University Biological Services Unit under a license from the Department of Health (Republic of Ireland) and with ethical approval granted from the University College Cork Ethics Committee. The methods applied in this study were carried out in accordance with the approved guidelines.

Peritoneal macrophages were collected from wild-type, TLR4- and TLR2-deficient mice by peritoneal lavage and incubated with DMEM containing 10% heat-inactivated FCS in 96- or 24-well plates (Falcon, Lincoln Park, NJ, USA) for 90 min to remove non-adherent cells as described previously^57^. BMMs were isolated from the femurs of wild-type, TLR4- and TLR2-deficient mice and cultured in DMEM containing 20% heat-inactivated FCS, penicillin (100 U/ml), streptomycin sulfate (100 μg/ml) and supplemented with 10 ng/ml recombinant mouse macrophage-CSF (R&D Systems, Minneapolis, MN, USA) for 7 d at 37 °C in a humidified 5% CO_2_ atmosphere as described previously^57^. The purity of both isolated peritoneal macrophages and BMMs was >95%, as confirmed by FACScan analysis of the positive staining for F4/80 Ag with a rat anti-mouse F4/80 Ab (Serotec, Oxford, UK).

### Human monocyte isolation and cultures

Human peripheral blood mononuclear cells (PBMCs) were separated from the whole blood of healthy volunteers by differential gradient centrifugation over endotoxin-free Ficoll-Paque Plus (Amersham Biosciences, Amersham, UK) and human monocytes were separated from PBMCs through differential gradient centrifugation over iso-osmotic Percoll (GE Healthcare, Buckinghamshire, UK) as described previously^58^. Purity of the isolated monocytes was >90% as confirmed by FACScan analysis of the CD14-positive cells. All experiments performed on human volunteers were approved by the University College Cork Teaching Hospitals Ethics Committee. The methods applied were carried out in accordance with the approved guidelines. Written informed consent was obtained from each volunteer before venesection. Isolated human monocytes were cultured in RPMI 1640 supplemented with 10% heat-inactivated FCS at 37 °C in a humidified 5% CO_2_ atmosphere.

### Cytokine measurement and cell viability assay

Peritoneal macrophages and BMMs isolated from wild-type, TLR4- and TLR2-deficient mice were pre-stimulated either with various doses of mMrp8 for 18 h or with 1 μg/ml mMrp8 for various time periods and re-stimulated with 5 μg/ml mMrp8, 100 ng/ml LPS, 100 ng/ml BLP, 5 × 10^4^ CFU/ml heat-killed *S. typhimurium* or 7.5 × 10^4^ CFU/ml heat-killed *S. aureus* for 6 h. Isolated human monocytes were pre-stimulated either with various doses of hMrp8 for 12 h or with 0.1 μg/ml hMrp8 for various time periods and re-stimulated with 5 μg/ml hMrp8, 100 ng/ml LPS, 100 ng/ml BLP, 5 × 10^4^ CFU/ml heat-killed *S. typhimurium* or 7.5 × 10^4^ CFU/ml heat-killed *S. aureus* for 6 h. BMMs isolated from wild-type, TLR4-deficient or TLR2-deficient mice were pre-stimulated with 1 μg/ml mMrp8 for 18 h and re-stimulated with 100 ng/ml LPS or 100 ng/ml BLP for 6 h. Cell-free supernatants were collected and stored at −80 °C until analysis. TNF-α and IL-6 concentrations in the supernatants were assessed by ELISA (eBioscience, Hatfield, UK).

Cell viability was assessed using Resazurin assay, as described previously^59^.

### Transient transfection

HEKhTLR4 cells stably transfected with human TLR4, CD14 and MD2 cDNA constructs purchased from Invivogen (San Diego, CA, USA) and HEKhTLR2 cells stably transfected with a human TLR2 cDNA construct (a gift from Dr. Evelyn A. Kurt-Jones, University of Massachusetts Medical School, Worcester, MA, USA) were maintained in DMEM supplemented with 10% FCS and G418 (0.5 mg/ml). HEKhTLR4, HEKhTLR2 and HEK293 cells plated in 24-well plates (Falcon) at 1 × 10^5^ cells/well were transfected with 400 ng of NF-κB-driven firefly luciferase plasmid (pNF-κB-Luc) (Clontech, Mountain View, CA, USA) and 10 ng of CMV promoter-driven *Renilla* luciferase plasmid (phRL-CMV) (Promega, Madison, WI, USA) for 16 h. After transfection, cells were stimulated with 5 μg/ml hMrp8, 100 ng/ml LPS or 100 ng/ml BLP for 6 h. Luciferase activity was determined using the dual-luciferase reporter assay system (Promega). Transfection efficiency was normalized in all experiments with simultaneously measured *Renilla* luciferase activities.

### Bacterial uptake, ingestion, and intracellular bacterial killing

Bacterial uptake, phagocytosis, and intracellular bacterial killing were determined as described previously^60^,^61^. Briefly, isolated human monocytes were pretreated with either culture medium (naive) or 0.1 μg/ml hMrp8 (Mrp8-tolerised) for 12 h and further incubated either with FITC-conjugated *E. coli* (Molecular Probes, Eugene, OR, USA) or with FITC-conjugated *S. aureus* (Molecular Probes) at a ratio of 1:50 (monocyte:bacteria) at 37 °C for 30 min. Bacterial uptake by naive and Mrp8-tolerised monocytes was assessed by FACScan analysis. Bacterial phagocytosis was further determined after the external fluorescence of the bound, but non-ingested, bacteria was quenched with 0.025% crystal violet (Sigma-Aldrich). To determine intracellular bacterial killing, naive and Mrp8-tolerised monocytes were incubated with live *S. typhimurium* or live *S. aureus* (monocyte:bacteria = 1:50) at 37 °C for 20 min to allow bacterial phagocytosis to take place. After this 20-min incubation period, the co-culture of monocytes and bacteria was incubated with 100 μg/ml gentamycin (Hospira, Lake Forest, IL, USA) at 37 °C for further 30 min to kill the extracellular non-ingested bacteria. After monocytes were lysed, the intracellular bacterial load was determined by incubation of serial 10-fold dilutions of the lysates on tryptone soy agar (Merck) plates at 37 °C for 24 h. The intracellular bacterial load was considered as an inverse measurement of intracellular bacterial killing.

### Western blot analysis

Isolated human monocytes pre-incubated with culture medium or 0.1 μg/ml hMrp8 for 12 h were stimulated with 5 μg/ml hMrp8 or 10 ng/ml LPS for various time periods, washed with ice-cold PBS and lysed on ice in cell lysis buffer (Cell Signaling) supplemented with 1 mM PMSF and protease inhibitor cocktail (Roche Diagnostics GmbH, Mannheim, Germany). The resultant lysates were centrifuged and supernatants containing the cytoplasmic proteins were collected. Protein concentration was measured using a micro BCA protein assay (Pierce, Rockford, IL, USA). Equal amounts of protein extracts were separated on SDS-polyacrylamide gels and *trans*-blotted onto polyvinylidene difluoride membranes (Schleicher & Schuell, Dassel, Germany). Membranes were blocked for 1 h at room temperature with PBS containing 0.05% Tween-20 and 5% nonfat milk and probed overnight at 4 °C with the appropriate primary mAbs anti-phosphorylated p38 at Tyr^182^ or anti-phospho-IκBα at Ser^32^. Blots were then incubated with the appropriate HRP-conjugated secondary Abs (Dako, Cambridge, UK) at room temperature for 1 h, developed with SuperSignal chemiluminescence substrate (Pierce) and captured with LAS-3000 imaging system (Fujifilm, Tokyo, Japan).

### Assessment of total IκBα, phosphorylated NF-κB p65 and p38

Human monocytes pre-incubated with culture medium or 0.1 μg/ml hMrp8 for 12 h were stimulated with 5 μg/ml hMrp8 or 100 ng/ml LPS for various time periods. Cells were fixed with phosflow fix buffer (BD Biosciences, San Jose, CA, USA), permeabilized with phosflow perm buffer (BD Biosciences), and stained with Alexa Fluor 488-conjugated mouse anti-human IκBα (Cell Signalling), PE-conjugated mouse anti-human phosphor-p65 (pS529) (BD Biosciences) or PE-conjugated mouse anti-human phosphor-p38 (pT180/pY182) (BD Biosciences) mAbs. Alexa Fluor 488- or PE-conjugated anti-human isotype-matched mAbs (Cell Signalling) (BD Biosciences) were used as the negative control. FACScan analysis was performed from at least 10,000 events for detecting total IκBα, phosphorylated NF-κB p65 and p38 using CellQuest software (BD Biosciences). The mean fluorescence intensity (MFI) was used to determine fold changes of total IκBα, phosphorylated NF-κB p65 and p38 between naive and Mrp8-tolerised monocytes after re-stimulation with hMrp8 or LPS.

### LPS-induced endotoxin shock and CLP-induced polymicrobial sepsis models

Pyrogen-free, 8- to 10-wk-old C3H/HeN mice received intraperitoneal injection of 200 μl PBS (naive), 50 μg/mouse mMrp8 (Mrp8-tolerised) or 5 mg/kg LPS (LPS-tolerised) 24 h before septic challenges. Endotoxin shock was induced by the intraperitoneal injection of a lethal dose of LPS (30 mg/kg). Polymicrobial sepsis was induced by CLP as described previously^61^. Blood samples were collected at 90 min and 4 h post LPS injection or at 2 and 6 h post CLP, and serum TNF-α and IL-6 were assessed by cytometric bead array (BD Biosciences, San Jose, CA, USA). Survival was monitored for at least 7 days.

### Measurement of peritoneal leukocyte subpopulations

Peritoneal lavage was collected from naive and Mrp8-tolerised mice before and after CLP, and dual-stained with anti-Ly-6G (BD PharMingen, San Diego, CA) and anti-F4/80 (Serotec) mAbs conjugated with PE or FITC. PE- or FITC-conjugated anti-mouse isotype-matched mAbs (BD PharMingen) (Serotec) were used as the negative control. FACScan analysis was performed from at least 10,000 events for detecting the subpopulations of PMNs (Ly-6G-positive cells) and macrophages (F4/80-positive cells) using CellQuest software (BD Biosciences).

### Enumeration of bacteria in the blood and visceral organs

Bacterial counts were determined as described previously^60^,^61^. Briefly, naive and Mrp8-tolerised mice were culled at 12 and 24 h post CLP. Blood samples were obtained by retinal artery puncture, and the dissected liver and spleen were homogenized in sterile PBS. Serial 10-fold dilutions of heparinized blood and organ homogenates in sterile water containing 0.5% Triton X-100 (Sigma-Aldrich) were plated on brain heart infusion agar (BD Biosciences) and incubated for 24 h at 37 °C for determination of bacterial colony-forming unit (CFU).

### Statistical analysis

All data are presented as the mean ± SD. Statistical analysis was performed using the log rank test for survival, and Mann-Whitney *U* test or unpaired t test with Welch’s correction where appropriate, with GraphPad software version 5.01 (Prism, La Jolla, CA). Differences were judged to be statistically significant when the *p* value <0.05.

## Additional Information

**How to cite this article**: Coveney, A. P. *et al.* Myeloid-related protein 8 induces self-tolerance and cross-tolerance to bacterial infection via TLR4- and TLR2-mediated signal pathways. *Sci. Rep.* **5**, 13694; doi: 10.1038/srep13694 (2015).

## Supplementary Material

Supplementary Figures
