# Supplementary material for: Myeloid-related protein 8 induces self-tolerance and cross-tolerance to bacterial infection via TLR4- and TLR2-mediated signal pathways
Source: Sci Rep. 2015 Sep 2;5:13694. doi: 10.1038/srep13694 (PMC4642578; doi:10.1038/srep13694)
Supplement: Supplementary Figures [file srep13694-s1.pdf]

Myeloid-related protein 8 induces self-tolerance and cross-tolerance to bacterial infection via TLR4- and TLR2-mediated signal pathways

Andrew P. Coveney, Wei Wang, Justin Kelly, Jing Hua Liu, Siobhan Blankson, Qiong Di Wu, H. Paul Redmond, and Jiang Huai Wang

Figure S1

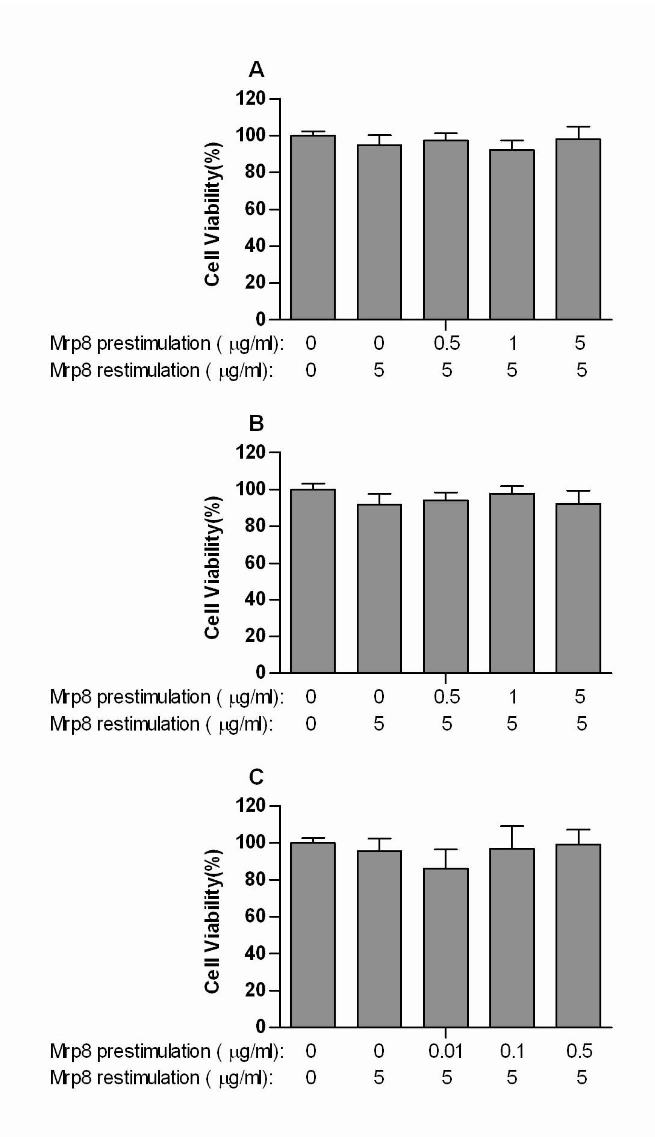

Fig. S1. Mrp8 pre-treatment does not cause cytotoxicity in both murine macrophages and human monocytes. Peritoneal macrophages (***A***) and BMMs (***B***) isolated from C3H/HeN mice were pre-stimulated with increasing doses of mMrp8 for 18 h and re-stimulated with 5 µg/ml mMrp8 for 6 h, whereas isolated human monocytes (***C***) were pre-stimulated with increasing doses of hMrp8 for 12 h and re-stimulated with 5 µg/ml hMrp8 for 6 h. Cell viability was assessed by Resazurin assay as described in the Materials and Method. Data are presented as mean ± SD of three independent experiments and each experiment was carried out in triplicate (***A*** and ***B***) or in quadruplicate (***C***).

Figure S2

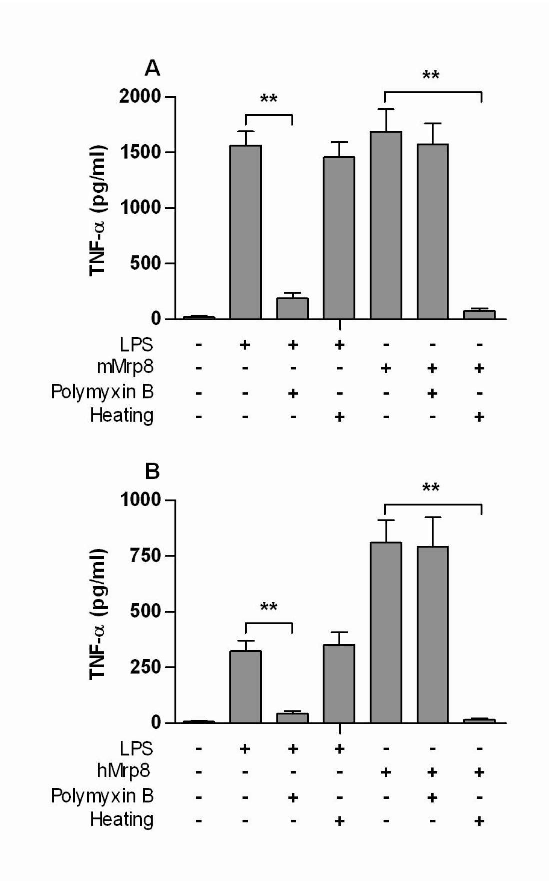

Fig. S2. Inactivation of Mrp8 by heating abrogates Mrp8-stimulated TNF-α release from murine BMMs and human monocytes. Isolated BMMs from C3H/HeN mice (***A***) and human monocytes (***B***) were pre-incubated with polymyxin B (25 µg/ml) and further stimulated with 100 ng/ml LPS and 5 µg/ml mMRP8 (***A***) or 10 ng/ml LPS and 5 µg/ml hMrp8 (***B***) for 6 h. In addition, LPS, mMrp8 and hMrp8 were heated at 80°C for 30 min before stimulation (heating). TNF-α concentrations in the culture supernatants were assessed by ELISA. Data are presented as mean ± SD of five independent experiments and each experiment was carried out in duplicate. **p<0.01 compared with murine BMMs stimulated with 100 ng/ml LPS or 5 µg/ml mMrp8 (***A***) (unpaired t test with Welch's correction); **p<0.01 compared with human monocytes stimulated with 10 ng/ml LPS or 5 µg/ml hMrp8 (***B***).
